# Nursing Students’ Perceptions of Learning Through a Service Learning Programme with Older Adults Living in Poverty in a High-Income Country: A Descriptive Qualitative Study

**DOI:** 10.3390/healthcare12242493

**Published:** 2024-12-10

**Authors:** Cristina Guerra-Marmolejo, Wladimir Morante-García, Ousmane Berthe-Kone, Anabel Chica-Pérez, Iria Dobarrio-Sanz, José Granero-Molina, Ana Belén Ortega-Avila

**Affiliations:** 1 Departamento de Enfermería, Universidad de Málaga, 29071 Málaga, Spain; cguerra@uma.es (C.G.-M.); anaortavi@uma.es (A.B.O.-A.); 2 Médicos del Mundo Andalucía, 04001 Almería, Spain; wladimir.morante@medicosdelmundo.org; 3 Nursing, Physiotheraphy and Medicine Department, University of Almería, 04120 Almería, Spain; ob053@inlumine.ual.es (O.B.-K.); jgranero@ual.es (J.G.-M.); 4 Unidade de Investigação em Ciências da Saúde: Enfermagem, Escola Superior de Enfermagem de Coimbra, 3004-011 Coimbra, Portugal; sanziria@esenfc.pt; 5 Facultad de Ciencias de la Salud, Universidad Autónoma de Chile, Santiago 7500000, Chile

**Keywords:** service learning, older adults, poverty, qualitative research, nursing students

## Abstract

Background: Living in poverty negatively affects the biopsychosocial health of older adults. Nursing students need to develop competence to promote health and self-care behaviours amongst older adults living in poverty in high-income countries. Aim: To explore nursing students’ perceptions of a service learning programme aimed at promoting health and self-care among older adults living in poverty in a high-income country. Methods: A descriptive qualitative study was conducted with 37 nursing students recruited from a university in Southeastern Spain. Participants completed a service learning programme in which they conducted preventive home visits to older adults living in poverty. Data were collected through in-depth interviews and analysed using a reflexive thematic analysis. Results: Three key themes were developed: (1) service learning as a different way of learning, (2) as an active learning approach to bridge the theory–practice gap, and (3) to provide insight into an unknown side of nursing. Conclusions: By providing opportunities for active, experiential learning in real-world contexts, service learning was perceived by the nursing students as a methodology that helps to bridge the gap between theory and practice. Participating in a service learning programme with older adults living in poverty not only fosters emotional engagement, reflective practice, and the development of essential non-technical skills in nursing students, but it also prepares them to advocate for older adults living in poverty in a high-income country.

## 1. Introduction

Available evidence suggests that living in poverty is an independent predictor of mortality [1]. Poverty goes beyond having a low income [2], and it is linked to social exclusion, which occurs as a result of social behaviours and institutional practices and policies [3]. In Europe, one in seven older adults (people aged 65 and above) are at risk of living in poverty [4]. In Spain, the proportion of older adults at risk of poverty or social exclusion reaches almost 21% [5]. Furthermore, we know that in Andalusia (i.e., Spain’s most populated region), 7.9% of older adults live in severe poverty [6]. In this context, it is important that nurses are able to develop interventions that address the specific needs of older adults living in poverty [7].

Nursing interventions for older adults living in poverty should aim to facilitate access to healthcare services, promote health, and foster self-care [8]. However, previous studies suggest that nurses continue to face barriers to implementing quality care for older adults living in poverty [9]. These barriers include a lack of theoretical and practical knowledge of how to care for older people living in poverty, insufficient training in specific clinical settings [10], and a lack of experience in working in socially deprived contexts [11]. It is therefore recommended that undergraduate nursing programmes include active educational interventions for students to be able to acquire the necessary competence to promote health and foster self-care amongst older adults living in poverty [12,13]. In this regard, service learning could be an interesting educational approach, as it has shown positive results for both nursing students and the vulnerable population involved in such interventions [14,15].

Service learning is an experiential learning approach that integrates academic learning with community service, which addresses identified community needs while combining learning objectives with community benefits [16]. This approach allows students to apply theoretical knowledge to real-world settings, fostering a reciprocal benefit for both the learners and the community involved [17,18]. As suggested by Kolb’s experiential learning theory [19], learning is a process that is facilitated by direct experience followed by reflection. Kolb’s cycle includes four stages: concrete experience, reflective observation, abstract conceptualization, and active experimentation [19]. This framework is particularly meaningful for service learning programmes as students engage in concrete experiences while working with vulnerable older adults. They then reflect on these experiences, conceptualize their learning, and apply it in future interactions with the community [19].

Recent studies show that service learning programmes involving nursing students have a positive impact on both the students and the community that participate in the programme [20]. Indeed, service learning programmes have been shown to improve nursing students attitudes towards older adults [16], as well as help them to develop interpersonal, reflective, critical thinking [21], and problem-solving skills [22]. In fact, service learning programmes lead to improved clinical reasoning skills, which suggests that their inclusion in undergraduate nursing programmes may be beneficial [23]. Previous studies have reported positive outcomes after implementing service learning programmes with medical students in primary care [24], physical therapy students in paediatric settings [25], pharmacy students in community care [22], and nursing students with older adults [26]. Nonetheless, there is a lack of studies focusing on analysing the educational effects of service learning programmes on nursing students’ competence to promote health and foster self-care amongst older adults living in poverty in a high-income country. The aim of this study was to explore nursing students’ perceptions of learning through a service learning programme with older adults living in poverty in a high-income country.

## 2. Materials and Methods

### 2.1. Design

This study followed a descriptive qualitative design, which provides participants with insight into a given phenomenon [27]. These studies require less abstraction in data analysis, making them an excellent choice for research in healthcare settings [28]. The consolidated criteria for reporting qualitative research (COREQ) were followed to write this manuscript [29].

### 2.2. Participants, Context, and Description of the Service Learning Programme

This study was carried out at a university in Southeastern Spain. Inclusion criteria for participation in the study were as follows: (1) being enrolled in the module ‘Elderly Care’ of the Nursing Degree programme; and (2) having completed the service learning programme focused on home visits for health and self-care promotion amongst older adults living in poverty. Participants were recruited through purposive sampling. Fifty students were invited to participate by one of the researchers (who had no professional or personal relationship with them) via a phone call in which the study’s title and purpose were stated. Twelve students declined due to lack of time and 1 did not respond. The participants who expressed their wish to participate in the study were contacted again to arrange an appointment outside of their lessons and clinical placement hours for their interview.

The service learning programme was part of the ‘Elderly Care’ module included in the third year of a Nursing Degree programme in a Southeastern Spanish university, and it was organised in collaboration with ‘Doctors of the World Andalusia’, a non-governmental organisation that has coordinated other intervention programmes with older people living in poverty. The service learning programme was a mandatory assignment as it was associated with a specific competence the nursing students needed to achieve before completing the module. The nursing students participating in the service learning programme had to complete four visits (one visit a month for the four months of the module) that were each approximately 3 h long. They took place in the older adults’ homes and aimed at promoting health and fostering self-care behaviours. The students were accompanied and supervised by a qualified member of staff from ‘Doctors of the World Andalusia’. All nursing students participating in the service learning programme had already completed the ‘Community Nursing Care’ and ‘Community Care Clinical Placement’ modules, which meant that they had been signed off as competent at promoting self-care through home visits. In addition, before commencing the service learning programme, the nursing students received 20 h of training on the same topics they would have to cover during their home visits: (1) how to assess the biopsychosocial needs of older adults living in poverty (5 h); (2) how to promote self-care for older adults living in poverty (5 h); (3) how to foster healthy eating amongst older adults living in poverty (5 h); and (4) how to offer care navigation support for older adults living in poverty within the study context (5 h). As part of their visits, the students also had the opportunity to accompany the older adults to the local supermarket to shop, which was funded by the knowledge transfer project that included the service learning programme.

### 2.3. Data Collection

Data collection took place between January and June 2023 through in-depth interviews that ranged in length from 40 to 52 min (mean duration = 43 min). The interviews were audio-recorded with the consent of the participants. An interview script was designed to allow nursing students to describe their perceptions in detail. The interview script was pre-rehearsed, and the interviewer was instructed to make the interviews resemble a natural conversation. During the interviews, the interviewer made notes on non-linguistic elements of communication. All interviews started with the question: ‘What did you think about the service learning activity with older people experiencing poverty?’ After 37 interviews had been conducted, the researchers considered that they had reached the point of theoretical saturation, and data collection was stopped. Theoretical saturation is reached when no new significant themes, concepts, or insights emerge from additional data collection [30]. In this study, saturation was achieved when subsequent interviews yielded repetitive information and did not provide additional relevant data.

### 2.4. Data Analysis

The interview recordings were transcribed and added into a hermeneutic unit in ATLAS.ti.23 to be analysed together with the interviewers’ annotations. Data analysis was conducted following the 6-step procedure of reflexive thematic analysis in [31]. (1) Familiarisation with the data: researchers read all transcripts and extracted their general meaning, then re-read and wrote annotations in ATLAS.ti. (2) Systematic coding of the data: researchers selected the most important quotes and assigned meaningful codes to them. The coding was performed by two trained researchers. To ensure consistency in the coding process, any discrepancies in the initial coding were discussed and resolved by reaching a consensus. (3) Generation of themes: the codes were grouped into initial sub-themes and these were grouped into themes. (4) Development and review of the themes: this step was carried out by checking both that the themes developed were consistent with the codes and that the quotes matched the assigned codes. (5) Refining themes: the initial themes and sub-themes were refined, defined, and assigned a definite name. (6) Report writing: the most relevant quotes were selected to be included in the report so that the analysis was clearly linked back to the research question. While inter-coder reliability was not calculated using statistical methods, the iterative process of discussion and agreement between the coders ensured the consistency and validity of the coding scheme.

### 2.5. Rigour

The methodological rigour of this study was maximized by taking actions to optimise its credibility, transferability, dependability, and confirmability [32]. Credibility: In order to ensure credibility, the research team was composed of expert academics, clinicians, and nursing students. In addition, the person who conducted the interviews was a postgraduate nursing student who had interacted with the participants in the past over a long period of time and had built a trusting relationship with them. In addition, the results were confirmed by the participants and some of their quotations were included in the report. Transferability: The participants’ characteristics, their context, and the service learning programme were described in detail. Dependability: A detailed description of the methodology has been provided so other researchers can replicate the study. Confirmability: we returned the data analysis to the study participants who confirmed that the themes and subthemes reflected their perceptions of learning through a service learning programme with older adults living in poverty in a high-income country.

### 2.6. Ethical Considerations

This study was conducted in accordance with the ethical principles of the Declaration of Helsinki. Participants were informed of the team’s commitment to data processing, which respected anonymity and confidentiality. They were also informed of the purpose of the study and the voluntary nature of their participation. Informed consent was obtained from the participants before starting data collection. The data were processed in accordance with the European and Spanish legislation on personal data protection. The study was approved by the Ethics and Research Commission of the Department of Nursing, Physiotherapy, and Medicine of the University of Almeria on the 6 October 2021, with protocol number EFM152/2021.

## 3. Results

A total of 37 nursing students with a mean age of 24.35 (SD = 7.29; range = 21–55) participated in the study. Of these, 15 were women and 12 were men. All of them had completed a clinical placement in primary care (6 weeks). However, more than 35% of the participants had not had any experience in working specifically with older adults before entering the service learning programme. The most relevant sociodemographic characteristics of the participants are shown in Table 1.

We developed the following three themes that describe the nursing students’ perceptions of learning through a service learning programme for older adults living in poverty: [1] service learning as a different way of learning, [2] as an active learning approach to bridge the theory–practice gap, and [3] as a window to a lesser-known side of nursing. The three themes, together with their sub-themes and codes, are summarised in Table 2.

### 3.1. Service Learning as a Different Way of Learning

The participants perceived service learning as an active methodology in which they became the protagonists of the learning process and often compared it with the more passive role they adopt in other traditional educational approaches. Learning in contact with the natural environment made the participants feel that the cases were real and interesting, which led them to perceive the service learning programme with older adults living in poverty as an appropriate intervention to bridge the gap between theory and practice. The nursing students also made reference to how working with real older adults led them to experience a whole range of emotions that helped them to retain knowledge more easily, which they believe will help them in their future professional careers.

#### 3.1.1. Learning in Contact with the Natural Environment

The vast majority of the nursing students who participated in the service learning programme expressed how they did not learn by memorising the theoretical content of the module in an abstract way that does not reflect reality. Instead, the participants felt that having to apply the theoretical content to a real-life scenario helped them to understand the rationale behind the “what”, the “how”, and the “why” of the theory they had learned about in the classroom. The nursing students mentioned that they learned lessons that cannot be taught in books yet are just as important in the educational curriculum of nurses. First-hand experiences of culture shock or how neighbourly support networks work helped the participants to understand the complexity of the specific healthcare needs that older adults living in poverty in a high-income country actually have.

“*When you go there and see what the situation is, you really understand how difficult it is for these people to do things differently. Acquiring knowledge in a real-life environment has nothing to do with what is written in a syllabus.*”

(P1)

The nursing students who participated in the service learning programme with older adults living in poverty were immersed in a learning experience that was unfamiliar to them. All the participants referred to how the service learning programme intervention was focused on people’s real-life circumstances. This increased the nursing students’ motivation to learn new things that would answer their questions and help to meet the needs of the older adults they attended. Although the participants felt that the service learning programme helped them to link their previous experiences in primary care to what the older adults living in poverty needed, they also expressed how the programme helped them to discover the reality of how this population lives and how their environment plays a pivotal role in the way they manage their health.

“*From a learning point of view, I saw it as a more fun and different activity to usual classroom teaching and having contact with real patients is helpful because you see how they live and in what conditions*.”

(P15)

Furthermore, almost all participants referred to how they perceived the service learning programme to help them to assimilate concepts related to ageing that they had previously encountered and studied from a theoretical point of view. For example, many of the nursing students felt that being involved in real-life situations helped them to fully understand and identify the biopsychosocial changes that occur as we age and how the social determinants of health impact older adults’ care needs.

“*You can see first-hand how older people really experience difficulties in meeting their needs. And I learned how you can really develop interventions to help them. I learned first-hand what it is and how it is done. For example, nutritional support, which when you read about it in a book, you don’t really see the importance of it*.”

(P10)

#### 3.1.2. A Type of Learning Influenced by Emotions

According to the participants’ accounts, one of the most important educational needs of nursing students revolves around learning how to apply the knowledge acquired in the classroom to everyday nursing practice. Almost all of the nursing students who participated in the study talked about how the service learning programme helped them to extrapolate theoretical knowledge to real-life situations. Although this was considered challenging, the participants felt that linking someone’s health issues to their life story and understanding their current situation contributed to feeling invested in helping the older adult with whom they were working.

“*It is also a very good way to put all the theory that we see in class into practice because it is as if we put faces to the older adults, you see what their real problems are and it is impossible not to feel obliged to help however you can*.”

(P22)

Many of the nursing students who participated in the service learning programme defined the experience of carrying out home visits with older adults living in poverty as shocking and deeply moving. Interestingly, when the participants were asked to elaborate further on the feelings that their interactions with the older adults elicited, all of the nursing students linked their emotions (regardless of their nature) to deep learning. Being confronted with situations that were so different to what they knew led the nursing students to truly understand the older adults’ vulnerability and made them want to learn more to be able to help.

“*...later a neighbour came over who also had many financial and family problems and I still remember feeling very sad. It was really hard but it made me realise that I had three months to work hard and learn how to help these people*.”

(P12)

### 3.2. An Active Learning Approach to Bridge the Theory–Practice Gap

The nursing students who participated in this study believed that incorporating active learning approaches into undergraduate nursing programmes was essential for promoting the acquisition and retention of competence in health and self-care promotion in older adults living in poverty. According to our participants, the service learning programme not only helped them to acquire new knowledge and gave them the opportunity to put it into practice, but it also contributed to the development of the attitudinal dimension of competence. Participating in the service learning programme sparked changes in the nursing students’ attitudes towards older adults living in poverty and they even admitted to becoming more empathetic after the experience. All of the participants described how the service learning programme gave them the opportunity to acquire important skills that, in their opinion, cannot be learned in other contexts.

#### 3.2.1. “Learning What Being a Nurse Really Means”: Developing the Attitudinal Dimension of Competence

As the nursing students were confronted with real-life situations in which they had to promote the health of older adults living in poverty, they became aware of some stereotypes and negative attitudes they had displayed towards other people living in poverty in their past. The service learning programme made the nursing students reflect, and the vast majority of the participants openly admitted that the experience had helped them to realise that their attitudes towards people living in poverty were based on stereotypes. Working closely with older adults living in poverty contributed to breaking the nursing students’ prejudices towards, stereotypes of, and negative attitudes towards this population.

“*First of all, it [the service learning programme] is quite useful to deconstruct certain prejudices that we had about people belonging to a lower social class or living in an underprivileged neighbourhood*.”

(P32)

Another interesting finding of this study is how participating in the service learning programme reshaped the nursing students’ perception of what respect and compassion really mean for nurses. In this regard, the service learning programme with older adults living in poverty also promoted personal growth and emotional maturity in the nursing students, who became fully aware of the need to provide quality, patient-centred care regardless of an individual’s socioeconomic background. Reflecting on these practices allowed the participants to learn that a humble, respectful, and compassionate attitude could contribute to better collaborations with older adults living in poverty in a high-income country.

“*All this helps us to be able to train ourselves for the future and above all to be able to be more respectful and compassionate when treating people who you don’t really know and to be able to have a consolidated basis for humanized care, which will be very necessary for when we join the workforce as inexperienced health workers in the future*.”

(P30)

#### 3.2.2. An Opportunity to Acquire Skills That Are Not Learned in the Classroom

According to the participants, the holistic nature of the service learning programme provided an opportunity to truly understand why certain non-technical skills are important in clinical practice. The nursing students admitted that although they had talked about empathy, active listening, respect, and many other communication skills in the classroom environment, they had not really grasped their importance in health and self-care promotion interventions until they were confronted with the older adults living in poverty in the service learning programme. Many nursing students expressed how, in their experience, most non-technical skills cannot be acquired or retained by only working on them in a classroom setting. All the participants in our study were grateful for the opportunity that the service learning programme offered them to develop and consolidate important non-technical skills, such as managing silence, active listening, empathy, compassion, and ethical practice.

“*Knowing how to listen, managing silences, not judge, to empathise, to know how not to be repetitive with some questions that have already been asked or answered in the conversation to not be too direct, repetitive, etc.… All of that is very valuable learning*.”

(P11)

The participants perceived that the setting where the service learning programme took place helped them to acquire important skills in nursing practice. For example, many nursing students mentioned how carrying out the intervention in the older adults’ homes helped them to be more observant of the finer details regarding the person and their environment. This allowed them to include more information about social determinants of health in the decision-making process that determines how an intervention is implemented for a particular individual. In addition, the nursing students stated that they had learned how to perform an assessment interview more effectively and how active listening is fundamental to understanding the needs and concerns of older adults living in poverty in a high-income country.

“*Also, I really value having gained experience in carrying out a holistic assessment of the older adult, not only focusing on their physical condition, but also being able to check the person’s psychological, emotional, environmental, social, and economic situation*”.

(P13)

After participating in the service learning programme, the nursing students became aware of the need to connect with the older adults living in poverty on a deeper level. Many nursing students made reference to how they felt a deep connection with the people they visited by establishing a trusting relationship with them. The participants felt that the service learning programme gave them the opportunity to learn that it was not just about listening or treating the older adults with respect and empathy. The nursing students perceived that accompanying, emotionally supporting, and giving evidence-based advice to the older adults living in poverty forged the basis of their trusting relationship, which, in turn, maximised the effects of the health and self-care promotion intervention.

“*The service learning programme brings out your empathetic and humane side to establish a connection with the patient and help build a relationship of trust with them. This helped to make the conversations less uncomfortable with questions about the bathroom or financial resources, and helped the patient to open up to a stranger so we could work on their health taking into account their concerns, worries, and other personal problems...*”

(P19)

### 3.3. Service Learning as an Insight into an Lesser-Known Side of Nursing

Another interesting finding of our study was linked to how the nursing students perceived the service learning programme as a driving force for discovering a side of nursing practice they had never seen, talked about, or experienced before:

“*It was a very interesting assignment… I really liked it because nobody had talked about this before and I think it will be very useful for us as future nurses*”

(P13)

The participants expressed how having to deliver a health and self-care promotion intervention for older adults living in poverty led them to realise that advocacy for social justice was a very important aspect of nursing. As part of the service learning programme, the nursing students had to implement their intervention outside of healthcare institutions. Immersing themselves into the older adults’ environment forced the participants to come out of their comfort zone. Many nursing students referred to how not being in a healthcare setting while doing health and self-care promotion made them feel more vulnerable. However, they perceived it to have a positive impact on forging a relationship with the older adults living in poverty in a high-income country.

#### 3.3.1. The Nurse as an Advocate for Social Justice

The service learning programme helped the nursing students to realise that health promotion for older adults living in poverty is not just about telling them what to do and how to do it. Being involved in several home visits allowed the nursing students to experience first-hand the complexities of the biopsychosocial context in which older adults living in poverty find themselves.

“*The lives of the older adults we visited are very complicated with family issues and stuff like that. For me, this experience taught me that I have to be more conscious of the patient’s environment when doing health promotion*.”

(P31)

The participants perceived that if they truly wanted to have a positive impact on the way older adults living in poverty incorporated health-promoting behaviours into their daily routines, they had to empower them to take responsibility for their own actions and decisions. The nursing students involved in the service learning programme felt that this educational experience required them to learn more about how to empower older adults to take voluntary steps towards a health-promoting lifestyle. They identified patient empowerment as a potential strategy to increase the visibility of the population they worked with.

“*We are there to motivate, to guide, to empower people to make their own decisions and if we don’t work with humanity we are not doing anything. We cannot always be there and they need to make themselves heard and seen*.”

(P25)

Many of the nursing students made reference to how older adults living in poverty in a high-income country remain invisible not only to healthcare professionals, but also to society in general. Most of the participants recognized they were totally unaware of the living conditions that older adults in nearby neighbourhoods had to endure. By witnessing first-hand the difficulties faced by older adults living in poverty in a high-income country, the participants reflected on the role they will have as future nurses.

“*To be brutally honest, I had never thought about the existence of older people living in such bad conditions. That’s the one thing I take from this experience… From now on, I am sure I will never look at older people from these neighbourhoods the same way*.”

(P7)

Most nursing students expressed the importance of nurses becoming advocates for social justice and mentioned that they felt obliged to assume social responsibility as healthcare professionals:

“*How can you be a nurse and not help these people?*”

(P11)

This was perceived very positively by the participants who recognized that it has helped them to appreciate the diversity of settings in which a nurse can provide health care. Many nursing students stated that it is a nurse’s responsibility and duty of care to raise awareness and support people in situations of vulnerability.

“*On a professional level, I have learned that we have to do everything we can to help, and above all, I have learned that nursing is not only about technical procedures, but also about giving a lot of support, listening to vulnerable people, showing that we care, and making everyone aware of it, which differentiates us from many other professions*.”

(P14)

#### 3.3.2. Stepping out of the Comfort Zone of Institutionalised Nursing Care

At times, nursing interventions require nurses to come out of their institutions so they can reach vulnerable populations who have difficulties accessing healthcare services. Participating in the service learning programme led the nursing students to believe that tailoring a health and self-care promotion intervention to the specific needs of an older adult living in poverty was more likely to yield positive results, but it also required going beyond what hospitals or primary care centres offer.

“*...as a future professional, this has taught me that you have to be less judgmental and reach out and care for those who need it most and not those who are most comfortable for you to get to because they are closer or they come to see you*.”

(P4)

All the participants in the service learning programme made reference to how they had to spend time away from the institution that protects them and boosts their confidence. In the service learning programme, the nursing students had to enter the older adults’ own homes, which were their private space. When the participants had to go to the older adults’ homes, they went from feeling like the ones in charge to feeling like strangers in someone else’s home. Many nursing students reflected on this experience and admitted to having learned that nobody is in charge in the nurse–patient relationship, but rather two people work together towards the same objective.

“*You don’t have all the resources that you might have in the hospital and it is no longer the patient who is the ‘intruder’ who comes to the hospital, but rather me who was the ‘intruder’ who went to their home. And that changes the way you see it because you can’t give instructions there, and it should always be like that*.”

(P7)

Coming out of their comfort zone made the nursing students realise how out of place patients can feel in healthcare institutions. Becoming aware of their own fears and perceptions influenced the way they experience the intervention implemented in the service learning programme for older adults living in poverty in a high-income country. The nursing students perceived that the service learning programme helped them to immerse themselves in a situation in which they were as vulnerable as the older adults living in poverty. This was interpreted by the nursing students as a rewarding experience and one of the key elements that encouraged deep learning.

“*On a personal level, this experience has been very rewarding as I felt welcomed in a house that was not mine from the very first moment and also the family shared all their worries, fears, way of life, and thoughts with me and I helped them as much as I could. I feel this is something I will never forget*.”

(P26)

## 4. Discussion

The aim of this study was to explore nursing students’ perceptions of learning through a service learning programme with older adults living in poverty in a high-income country. The main results of our study suggest that nursing students not only perceived service learning as a different way of learning, but also as an active learning approach that helps to bridge the gap between theory and practice. Furthermore, nursing students considered service learning to be an insight into a side of nursing practice that was previously unknown to them.

The nursing students who participated in this study experienced service learning as a more active and hands-on approach to education compared to traditional learning methods, which concurs with the findings from previous research [33]. The participants described how being immersed in real-life situations fostered a sense of ownership over their learning, contrasting with the more passive roles often adopted in classroom-based learning. Working directly with older adults living in poverty allowed the nursing students to apply theoretical knowledge to practical situations, giving them a clearer understanding of the complexities and nuances of promoting health and self-care in underprivileged populations [34]. This finding supports the notion that service learning challenges the traditional structures of nursing education, in which passive learning can limit opportunities for students to develop the skills and critical thinking necessary to be a competent practitioner [33]. The participants’ reflections illustrated the value of experiential learning within real-world environments, suggesting that nursing education could benefit from a stronger emphasis on such methodologies. Previous research also highlights the importance of active learning for skills development and the acquisition of competence, particularly when students are exposed to the real-life contexts of vulnerable populations [35]. Moreover, the service learning programme allowed the nursing students to their integrate knowledge, skills, and attitudes to solve problems in situ, which is a crucial element of competence for health promotion and self-care amongst older adults living in poverty. Our results also point towards how learning in a service learning programme was strongly reinforced by emotional experiences [36,37]. Many participants described the emotional impact of engaging with older adults living in poverty, emphasizing how these emotions, whether positive or challenging, facilitated deeper learning. This aligns with previous findings suggesting that emotions play a pivotal role in the internalization of knowledge and the development of empathy among healthcare professions [38]. The nursing students who participated in the service learning programme reported how the emotional connection they developed with the older adults helped them to retain knowledge more effectively and increased their motivation to learn [39]. As some participants remarked, putting a human face to the theory learned in class transformed their abstract knowledge into something deeply personal and relevant. This emotional engagement was likely to have contributed to the students’ ability to link theory to practice more effectively. In line with another study’s findings [38], deep learning occurs when knowledge is mediated by emotions, values, and empathy, suggesting that service learning can foster compassion—a critical element of nursing practice that may not be as easily developed in a traditional classroom setting [40,41].

Service learning was perceived by the participants as a powerful tool for bridging the theory–practice gap, a well-documented challenge in nursing education [42]. The nursing students highlighted how the programme allowed them to apply their theoretical knowledge to real-life situations, thereby solidifying their understanding of key concepts in health and self-care promotion amongst older adults. The nursing students noted that this experiential learning approach not only enhanced their knowledge but also promoted the development of the attitudinal dimension of competence. This is particularly important as nursing educators often struggle to teach the attitudinal dimension of competence effectively, which is critical for providing compassionate, patient-centred care [33,43]. The service learning programme prompted nursing students to critically reflect on their own attitudes towards older adults living in poverty. Many participants admitted that they held stereotypes or negative attitudes toward people living in poverty prior to the programme. However, through their interactions with the older adults, they became more aware of these biases and worked to overcome them. This is consistent with findings from other studies, which have shown that service learning can promote meaningful reflection [44], reduce prejudice, and promote more positive attitudes toward underserved populations [45]. Moreover, the service learning programme helped the nursing students to develop key professional values, such as respect, compassion, and humility. The participants recognized that providing high-quality care requires not only technical skills, but also the ability to engage with patients on a personal level. This aligns with the conceptual model of human caring, which emphasizes the importance of caring relationships in nursing practice [43]. The service learning programme gave the students the opportunity to develop these relational skills in a way that traditional classroom learning could not. In addition to fostering the development of the attitudinal domain of competence, the service learning programme also helped the nursing students develop non-technical skills, such as empathy, active listening, and communication. These skills are often difficult to teach in a classroom setting but are essential for effective nursing practice [46]. The participants in this study recognised the value of these skills and appreciated the opportunity to practice them in a real-world context. These findings are consistent with previous research, which has shown that service learning can help nursing students develop the soft skills necessary for patient-centred care [47,48].

Another important finding of the service learning experience was that it allowed the nursing students to discover a side of nursing practice they had not previously encountered. Through their interactions with older adults living in poverty, the nursing students began to recognise the broader social responsibilities of their profession, including advocacy for social justice. This aligns with the recent literature that highlights the critical role of nurses as advocates for underprivileged populations [45,49]. The nursing students in this study described how the experience of the service learning programme challenged their preconceived notions about poverty and healthcare. Many participants acknowledged that prior to the service learning programme, they had been unaware of the extent to which social determinants, such as housing, household income, and access to healthcare, affected the health of older adults in their community. By stepping outside the institutional settings in which they were used to carrying out their clinical placements, the nursing students gained a more comprehensive understanding of how nurses can play an important role in advocating for social justice and addressing healthcare disparities [50,51]. Participating in the service learning programme also required the nursing students to step out of their comfort zone. By working directly with older adults in their homes rather than in traditional healthcare institutions, the students were forced to confront their own vulnerabilities and reassess their roles as future healthcare providers. Concurring with other studies [39], many participants reported that entering the private space of older adults made them feel like intruders, which led to a shift in the power dynamics they were accustomed to in hospitals or clinical settings. This shift in perspective allowed the nursing students to understand that nursing is not just about technical expertise, but also about building trusting relationships with patients based on mutual respect and empathy. This lesson was seen as particularly valuable, as it helped students recognise that patient-centred care requires nurses to adapt their approach based on the unique needs and circumstances of the individuals for whom they are caring [52,53]. The students’ experiences of feeling vulnerable in unfamiliar environments mirrored the vulnerability often felt by patients in healthcare institutions, helping them develop a deeper sense of empathy and compassion for older people living in poverty in a high-income country.

The results of this study align with the key principles of kolb’s experiential learning theory [19], which emphasizes that effective learning occurs through a continuous cycle of experience, reflection, conceptualization, and action. In this context, nursing students learned through concrete experiences of working directly with older adults living in poverty, corresponding to Kolb’s concrete experience stage [54]. This immersion in real-world settings allowed them to reflect on their experiences, which is a crucial part of reflective observation; the participants described how these interactions helped them question and transform their attitudes toward older adults and poverty. Furthermore, abstract conceptualization occurred when the students connected these experiences with theoretical concepts learned in class, allowing them to gain a deeper understanding of the complexities involved in promoting health and self-care among vulnerable populations. Finally, through active experimentation, students were able to apply and practise their newly acquired knowledge, thus enhancing their technical and emotional skills in real-life contexts. This cycle of experiential learning fostered the development of essential competencies such as empathy, problem solving, and ethical decision making, which are critical to patient-centred nursing practice.

### Limitations

Our study has some limitations. Although the sample is representative of the nursing student population in our context, it is not heterogeneous. All the participants were Spanish and Caucasian. The cultural homogeneity of our sample may limit the applicability of our findings to more diverse nursing populations, as learning perceptions and experiences could differ significantly across cultural and ethnic backgrounds. The participation of a more heterogeneous group of students in the sample, i.e., people of different nationalities from different cultures, could have yielded different results. Another limitation refers to the fact that, for organisational reasons, only one interview could be conducted at the end of the programme. Conducting multiple interviews throughout the service learning programme and complementing them with focus groups could have allowed us to delve deeper into the nursing students’ perceptions of learning at different stages. Furthermore, observing the students in the context in which the visits took place could have enriched our results. In fact, the research team is already working on the design of a service learning programme for older women living in poverty who are the main family caregiver for a relative. As part of this study, nursing students’ skills will be assessed through an objective standardized clinical evaluation and their experiences will be explored through several in-depth interviews and participant observations. Lastly, we recommend future research is conducted in socially deprived neighbourhoods in other cities with different socio-economic characteristics and different health and social support systems.

## 5. Conclusions

In conclusion, this study highlights the perceived impact that a service learning programme can have on the way nursing students develop specific competencies related to promoting health and self-care amongst older adults living in poverty in a high-income country. By providing opportunities for active, experiential learning in real-world contexts, service learning is perceived by nursing students as a methodology that helps to bridge the gap between theory and practice. Participating in a service learning programme with older adults living in poverty not only fosters emotional engagement and reflective practice, but also promotes the development of essential non-technical skills, such as advocacy, cultural competence, and social justice awareness, which could be crucial for the success of health promotion interventions with vulnerable populations. Moreover, participating in a service learning programme exposes nursing students to the broader social justice dimensions of nursing, preparing them to advocate for older adults living in poverty in a high-income country. Our findings suggest that service learning interventions should be incorporated into nursing curricula, as they could help students prepare for the complexities of professional nursing practice when caring for underprivileged populations. Considering these results, we recommend that nursing education programs integrate service learning experiences into their curricula to foster competencies such as advocacy, cultural competence, and reflective practice. These programs should be designed to provide students with real-world exposure to vulnerable populations, helping them to develop essential non-technical skills that are crucial for effective and compassionate nursing care. Future research should continue to explore the long-term impact of service learning on nursing competence and patient outcomes.

## Figures and Tables

**Table 1:** Sociodemographic characteristics of the participants (N = 37).

Participant	Age	Gender	Primary Care Placement	Elderly Care Experience
P1	21	Female	Yes	No
P2	25	Male	Yes	Yes
P3	20	Male	Yes	Yes
P4	21	Female	Yes	No
P5	20	Female	Yes	No
P6	27	Female	Yes	Yes
P7	21	Female	Yes	Yes
P8	21	Female	Yes	No
P9	22	Female	Yes	Yes
P10	20	Female	Yes	Yes
P11	21	Male	Yes	Yes
P12	21	Male	Yes	Yes
P13	26	Female	Yes	Yes
P14	21	Female	Yes	Yes
P15	32	Female	Yes	No
P16	27	Male	Yes	Yes
P17	33	Male	Yes	Yes
P18	21	Male	Yes	No
P19	46	Female	Yes	Yes
P20	21	Female	Yes	Yes
P21	22	Male	Yes	No
P22	21	Female	Yes	No
P23	21	Female	Yes	No
P24	21	Female	Yes	No
P25	22	Female	Yes	Yes
P26	21	Female	Yes	Yes
P27	21	Male	Yes	Yes
P28	23	Male	Yes	No
P29	27	Female	Yes	Yes
P30	21	Female	Yes	Yes
P31	23	Female	Yes	Yes
P32	21	Male	Yes	No
P33	21	Female	Yes	Yes
P34	21	Female	Yes	No
P35	32	Female	Yes	Yes
P36	55	Male	Yes	Yes
P37	21	Female	Yes	Yes

**Table 2:** Structure of themes, sub-themes, and units of meaning.

Themes	Subthemes	Units of Meaning
1. Service learning as a different way of learning.	Learning in contact with the natural environment.	Learning without studying, different ways of learning, first-hand experience of real needs, the influence of the environment, assimilating concepts.
	A type of learning influenced by emotions.	Applying theory to practice, the impact of experience on learning, learning through emotion.
2. An active learning approach to bridge the theory–practice gap.	“Learning what being a nurse really means”: developing the attitudinal dimension of competence.	Changing attitudes, reinforcing values, eliminating stereotypes, rethinking values, practising humanity.
	An opportunity to acquire skills that are not learned in the classroom.	Handling silences, communication skills, learning to observe, learning to conduct an interview, learning to listen, learning to care with respect, confidence, active listening, assessment of the environment, emotional strength, responsibility, solidarity, humility, holistic assessment, emotional support, accompaniment, establishing a relationship of trust, patience, empathy, health promotion.
3. Service learning as an insight into a lesser-known side of nursing.	The nurse as an advocate for social justice.	Empowerment of older adults, social responsibility, social justice.
	Stepping out of the comfort zone of institutionalised nursing care.	Coming out of one’s comfort zone, going beyond what institutions offer, being a stranger in someone else’s home, shared vulnerability.

## Data Availability

The data presented in this study are available on request from the corresponding author. The data are available from the Principal Investigator upon reasonable request. The transcripts were incorporated into an ATLAS.ti project for the analysis, and this software is necessary to access them. The recordings and transcripts have not been made public in a repository because the data of specific individuals are mentioned and their disclosure may compromise confidentiality.
